# Implementation of an asymptomatic bacteriuria assessment protocol for patients discharged from the emergency department

**DOI:** 10.1017/ash.2023.117

**Published:** 2023-02-27

**Authors:** Margaret R. Hitchins, Jeannette L. Bouchard, Christopher W. Ingram, Alison I. Orvin

**Affiliations:** 1 Department of Pharmacy, WakeMed Health and Hospitals, Raleigh, North Carolina; 2 University of North Carolina Eshelman School of Pharmacy, Chapel Hill, North Carolina; 3 Infection Control and Occupational Health, WakeMed Health and Hospitals, Raleigh, North Carolina

## Abstract

**Objective:**

We evaluated the impact of an asymptomatic bacteriuria (ASB) assessment protocol on the number of antibiotics prescribed for ASB after discharge from the emergency department (ED).

**Design:**

Single-center, before-and-after, retrospective cohort study.

**Setting:**

The study was conducted at a large community health system in North Carolina.

**Patients:**

Eligible patients were discharged from an ED without an antibiotic prescription and had a positive urine culture result after discharge from May through July 2021 (preimplementation group) and October through December 2021 (postimplementation group).

**Methods:**

Patient records were reviewed to determine the number of antibiotic prescriptions for ASB on follow-up call before and after implementation of an ASB assessment protocol. Secondary outcomes included 30-day admissions, 30-day ED visits, 30-day UTI-related encounters, and projected antibiotic days of therapy.

**Results:**

The study included 263 patients: 147 in the preimplementation group and 116 in the postimplementation group). There were significantly fewer antibiotic prescriptions for ASB in the postimplementation group (50% vs 87%; P < .0001). There were no differences in the incidence of 30-day admissions (7% vs 8%; P = .9761), 30-day ED visits (14% vs 16%; P = .7805), or 30-day UTI-related encounters (0% vs 0%, NA).

**Conclusions:**

Implementation of an ASB assessment protocol for patients discharged from the ED significantly reduced the number of antibiotic prescriptions for ASB on follow-up call without an increase in 30-day admissions, ED visits, or UTI-related encounters.

Asymptomatic bacteriuria (ASB) often represents colonization, but inappropriate treatment is common.^1–5^ Several studies have reported inappropriate treatment of ASB to range from 40% to 59% prior to intervention.^2–5^ The 2019 Infectious Diseases Society of America (IDSA) guidelines on the management of asymptomatic bacteriuria recommend against screening and treating asymptomatic bacteriuria except in patients who are pregnant or who have a urological procedure planned.^1^ Nevertheless, antimicrobials are commonly prescribed for ASB, resulting in overutilization of antimicrobial agents leading to increased resistance, adverse drug events, and cost.^6–9^

Due to these consequences, avoiding treatment for ASB has become a target for antimicrobial stewardship programs. Antimicrobial stewardship programs traditionally focus on inpatient care, but there is a growing effort to optimize outpatient antibiotic prescribing, including the emergency department (ED), due to the high rates of antibiotic prescriptions in the outpatient setting.^10,11^ Historically, antimicrobial stewardship efforts in the ED have been difficult due to high patient volume, frequent turnover, limited resources, and lack of follow-up.^10,11^

Several studies have utilized pharmacist intervention to successfully reduce overtreatment for ASB.^2,3,12^ One study reported that treatment of ASB in the ED was significantly decreased by 16% when utilizing pharmacist-driven education to physicians, advanced practice providers, and nurses.^2^ Many institutions have also utilized algorithms combined with education for physicians to reduce inappropriate treatment of inpatient ASB. However, many institutions are likely to use nurses or ancillary staff for culture reviews who may not have specific antibiotic stewardship training, often referred to as steward extenders. No known studies have evaluated the impact of implementing an evidenced-based algorithm for ED follow-up nurses to reduce inappropriate treatment of ASB.

Currently, our institutional pathway for ED culture follow-up involves nursing staff without specific antibiotic stewardship training to follow an order set and recommend treatment only based on the cultures and sensitivities. Notification of culture and sensitivity results is integrated into the electronic health system (EHS) and routed to the follow-up nurse. Dedicated follow-up nurses monitor the receipt of results during business hours from 7:00 a.m. to 5:00 p.m. The standing orders are not integrated into the EHS, but the follow-up nurses document in the EHS that they utilized the standing order set. Prior to implementation of this protocol, the standard process was to present all patients who were not prescribed an antibiotic and who had a positive urine-culture result after discharge to the ED attending for prescription decision. This process led to overprescribing of antibiotics for ASB on culture follow-up call. To reduce overall antibiotic prescribing for ASB, the standing orders were updated to require no action for patients discharged from the ED without an antibiotic prescription determined to have ASB. In this study, we assessed the impact of this protocol.

## Methods

### Design and patient selection

**Fig. 1.**
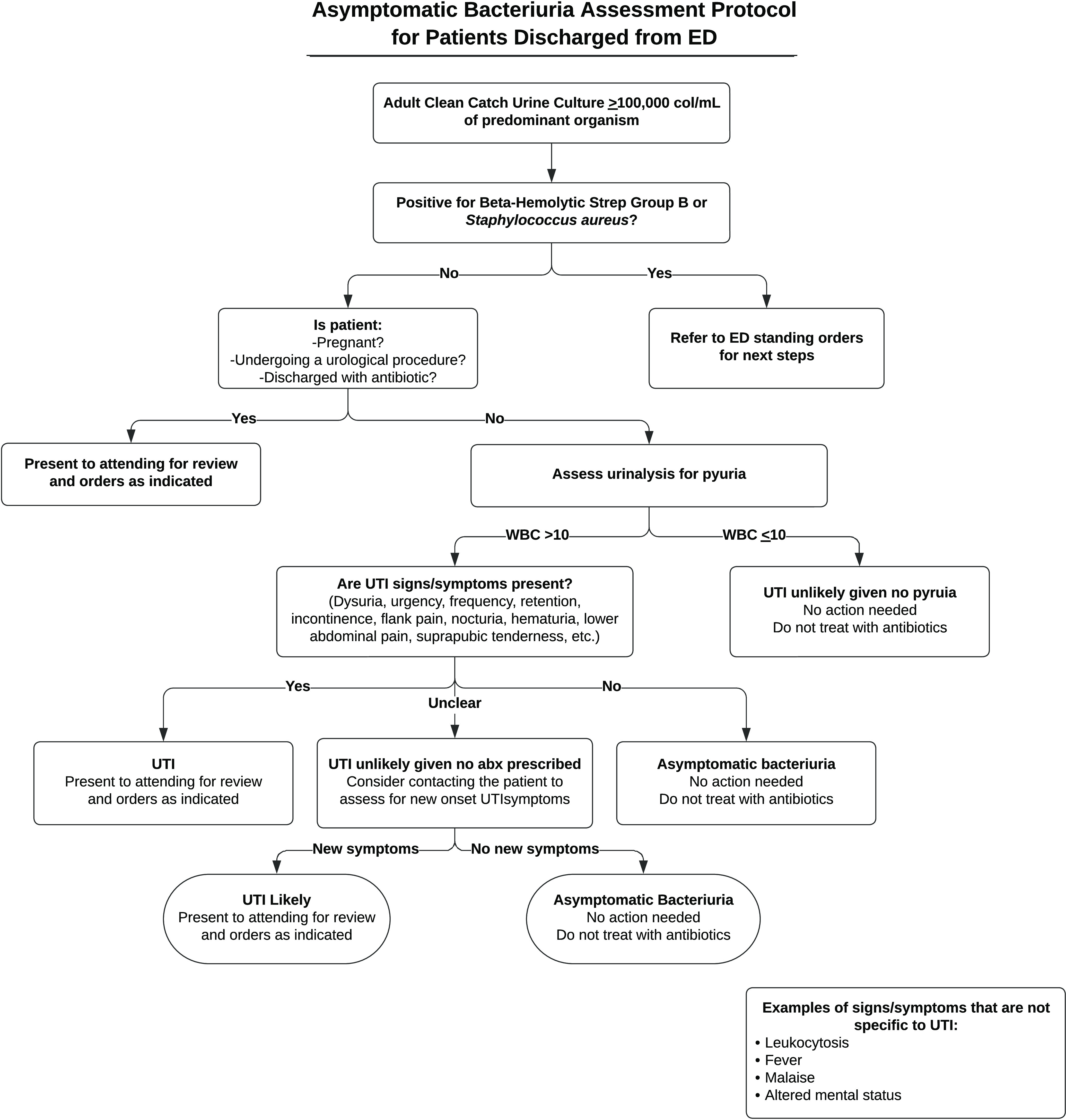
Algorithm for assessment of asymptomatic bacteriuria (ASB) utilized by emergency department follow-up nurses.

This investigation was a single-center, retrospective, before-and-after cohort study. This study included patients who were discharged from 1 of 6 WakeMed adult EDs without an antibiotic prescription and had a positive clean-catch urine culture positive result (≥100,00 colonies of single organism) after discharge. In 2021, there were ∼190,000 discharge encounters across the 6 EDs. We excluded patients who were pregnant, who were undergoing a urological procedure, or who had documented urinary symptoms or a urinary tract infection (UTI) diagnosis at the index ED visit. Patients were determined to have urinary symptoms based on the UTI signs and symptoms listed in Figure 1. Eligible patients were identified using EHS reports. The preimplementation group included patients discharged from any WakeMed ED from May 1 through July 31, 2021. The postimplementation group included patients discharged from any WakeMed ED from October 1 through December 31, 2021. The WakeMed Health and Hospitals Institutional Review Board considered this study to be a quality improvement initiative, and it was exempt from further review.

### Intervention

The ASB assessment protocol was implemented in August 2021 and consists of the ED follow-up nurses utilizing an algorithm to assess for ASB in adult patients (Fig. 1). Notably, cultures with *Staphylococcus aureus* were excluded because it can represent deep-seated infection.^13–16^ Consistent with current IDSA guidelines on the appropriate management of ASB, the protocol excludes patients who are pregnant or are undergoing a urological procedure.^1^ For patients who are included in the protocol, the follow-up nurses first evaluate the urinalysis for absence of pyuria given the high negative predictive value of nearly 90%.^17^ If the urinalysis shows >10 white blood cells (WBC) per high-power field (hpf), the follow-up nurses review the notes to see whether urinary symptoms have been documented. If the patient has urinary symptoms documented, the follow-up nurses present the patient to the ED attending for review and orders as indicated. These patients were excluded from study outcomes because they were considered not to have ASB. If unclear, they called the patient to clarify. If the patient has no urinary symptoms documented, the follow-up nurses do not present the patient to the ED attending and the patient does not receive treatment (Fig. 1).

### Outcomes and data collection

All data were collected using the electronic medical record. The following data were collected: age, sex, urine culture test (ie, reflex culture or not), indication for reflex criteria, organism, history of diabetes mellitus, spinal cord injury, dementia, nephrolithiasis, congenital urologic abnormality, and long-term care resident. If an antibiotic was prescribed, the antibiotic name and duration of therapy were collected. The primary outcome was the number of patients who received antibiotic therapy for ASB on follow-up call. Secondary outcomes included 30-day admission, 30-day ED visit, 30-day UTI-related encounter (ie, telephone call or office visit), and total days of antibiotic therapy.

### Statistical analysis

Continuous variables were described using median and interquartile range (IQR). Categorical variables were described using frequencies (%). Nominal variables were compared utilizing χ^2^ analysis or the Fisher exact test. Continuous variables were compared utilizing the Mann-Whitney *U* test. Statistical analyses were conducted using SAS JMP software, version 9 (SAS Institute, Cary, NC).

## Results

### Patient characteristics

**Fig. 2.**
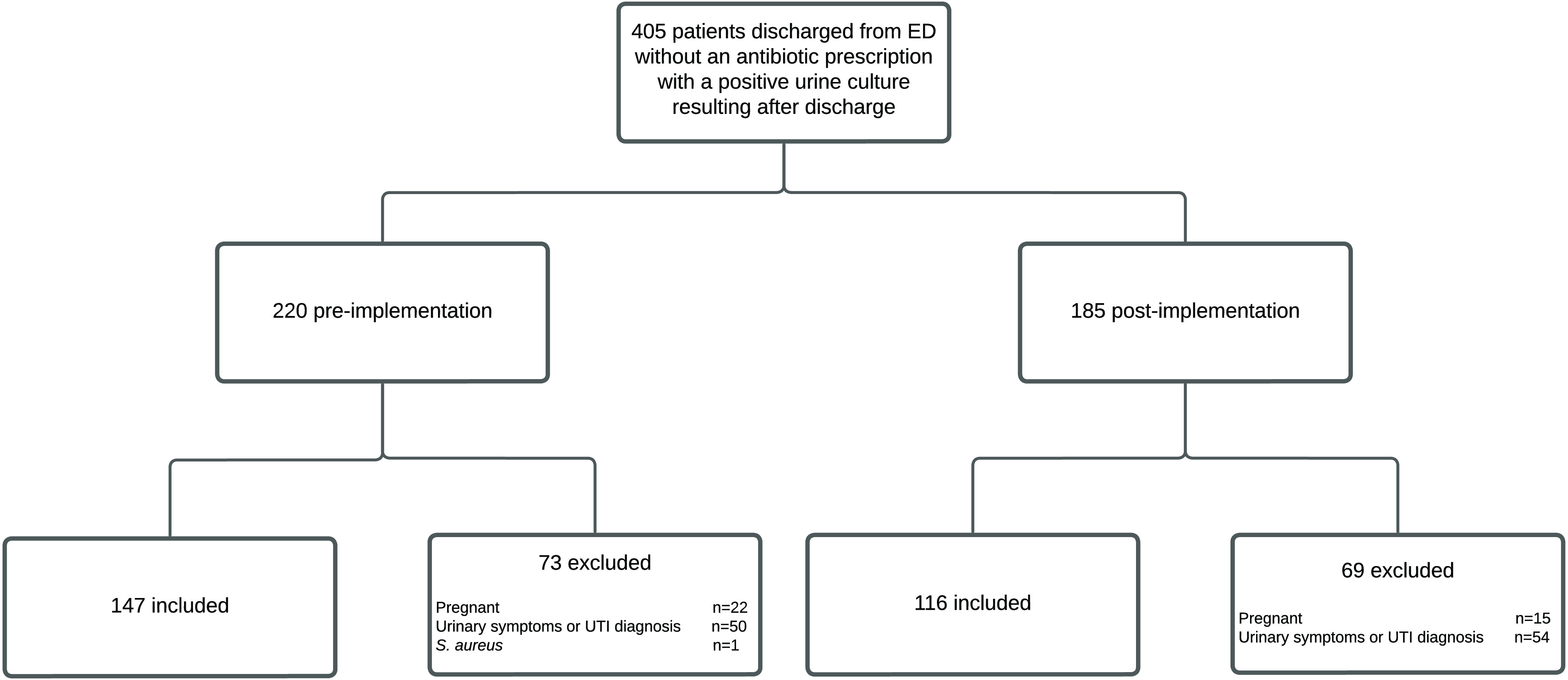
Inclusion and exclusion criteria.

During the study periods, 405 patients were discharged from a WakeMed ED without an antibiotic prescription who had a positive clean-catch urine-culture result after discharge. Of the 220 patients in the preimplementation group, 147 met eligibility criteria and were included in the analysis. Of the 185 patients in the postimplementation group, 116 met eligibility criteria and were included in the analysis. The primary reasons for exclusion included documented urinary symptoms or a UTI diagnosis and pregnancy (Fig. 2).

The baseline characteristics are shown in Table 1. The 263-patient study population was predominantly female (91%) with a median age of 42 years. The most common comorbidity was diabetes mellitus (21%). The most common urinary pathogen was *Escherichia coli* in both groups. There were significantly more *Klebsiella* spp in the postimplementation group (*P* < .01). Pyuria, defined as >10 WBC per hpf, was present in 24% of urine cultures in the preimplementation group and 26% of urine cultures in the postimplementation group (*P* = .7990).

**Table 1.** Baseline Patient Characteristics (N = 263)

Characteristic	Preimplementation Group (N = 147)	Postimplementation Group (N = 116)	*P* Value
Age, median y (IQR)	43 (31–73)	40 (27–69)	.2179
Sex, female, no. (%)	133 (90)	107 (92)	.6133
Diabetes mellitus, no. (%)	29 (20)	25 (22)	.7165
Spinal cord injury, no. (%)	2 (1)	0 (0)	.1262
Dementia, no. (%)	3 (2)	5 (4)	.2888
Nephrolithiasis, no. (%)	2 (1)	5 (4)	.1385
Congenital urologic abnormality, no. (%)	0 (0)	0 (0)	N/A
Long-term care resident, no. (%)	7 (5)	5 (4)	.8614
Urine pathogen, no. (%) *Escherichia coli* *Klebsiella* spp *Enterococcus* spp *Pseudomonas aeruginosa*	55 (37) 7 (5) 10 (7) 0 (0)	33 (28) 17 (15) 9 (8) 2 (2)	.1245 <.01 .7667 .0695
Urinalysis >10 WBC/hpf, no. (%)	36 (24)	30 (26)	.7990

Note. IQR, interquartile range; WBC, white blood cell count; hpf, high-power field; N/A, not available.

### Outcomes

**Fig. 3.**
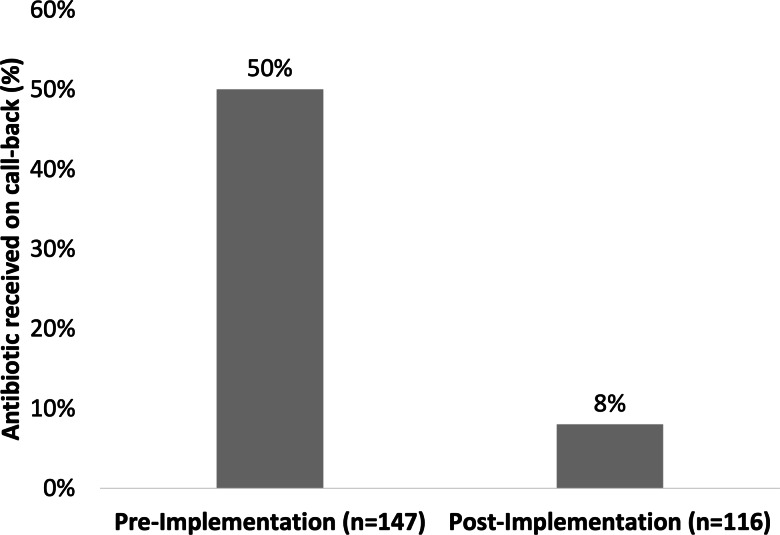
Primary outcome.

Overall, 74 patients received an antibiotic prescription for ASB after discharge from the ED in the preimplementation group and 9 in the postimplementation group (50% vs 8%; *P* < .0001) (Fig. 3). There was no difference between groups for 30-day admission and ED visits. No 30-day UTI-related encounters occurred, including UTI-related admissions, ED visits, and telephone encounters, in either group during the follow-up period. The projected antibiotic days of therapy was 492 days in the preimplementation group and 69 days in the postimplementation group (*P* < .0001). Secondary outcomes are shown in Table 2. A subgroup analysis of postimplementation 30-day secondary outcomes in patients who received antibiotic treatment compared with those who did not is shown in Table 3.

**Table 2.** Secondary Outcomes

Outcome	Preimplementation Group (N = 147)	Postimplementation Group (N = 116)	*P* Value
30-d admission, no. (%)	10 (7)	8 (7)	.9761
30-d ED visit, no. (%)	21 (14)	18 (16)	.7805
30-d UTI-related encounter, no. (%)	0 (0)	0 (0)	NA
Projected antibiotic days of therapy	492	69	<.001

Note. ED, emergency department; UTI, urinary tract infection.

**Table 3.** Subgroup Analysis of Postimplementation 30-Day Secondary Outcomes

Outcome	Treated (N = 9)	Untreated (N = 107)	*P* Value
30-d admission, no. (%)	2 (22)	6 (5)	.0428
30-d ED visit, no. (%)	1 (11)	17 (15)	.7452

Note. ED, emergency department.

## Discussion

Despite IDSA guidelines recommending against screening and treating ASB except in patients who are pregnant or who have a planned urological procedure, inappropriate treatment is common.^1–5^ This results in overutilization of antimicrobial agents, leading to increased resistance, adverse drug events, and cost.^6–9^ In this study, we evaluated the impact of an ASB protocol for patients discharged from the ED on the number of antibiotic prescriptions for ASB after discharge. Our results showed a significant decrease in antibiotic prescriptions for ASB without an increase in patient harm demonstrated by no change in 30-day admissions, 30-day ED visits, or 30-day UTI-related encounters.

The ED is a difficult setting in which to integrate antimicrobial stewardship principles due to the high patient volume and limited pharmacist availability for culture follow-up calls. Other studies have evaluated the impact of pharmacist driven culture follow-up call initiatives and physician education to improve ASB treatment in the ED. Shealy et al^12^ reported a significant reduction in time to recommendation and number of fluoroquinolone prescriptions when a pharmacist was utilized for culture follow-up call. Chowdhury et al^4^ reported a significant reduction in treatment for ASB from 47% to 15% (*P* = .04) through the use of multifaceted, hospital-wide education that included an algorithm.^4^ Similarly, a prospective chart review that utilized multifaceted education including an algorithm revealed significantly lower rates of inappropriate treatment in the intervention group (8% vs 48%; *P* < .001).^5^ At our institution, the use of pharmacists for culture follow-up calls is not achievable because the focus is on the higher-acuity patients in the ED during the day. For this reason, education and protocol optimization were focused on the nurses who conduct culture review. Limited data are available regarding culture follow-up call protocols in the ED for non–pharmacy-trained staff. Our study highlights an important and easily implemented protocol for nonpharmacy staff to act as steward extenders and partake in antimicrobial stewardship efforts. Additionally, our protocol is relatable to the many institutions that are unable to dedicate pharmacy services to culture follow-up calls.

This study had several limitations. First, only patients who were discharged from an ED without an antibiotic prescription were included to simplify the inclusion and exclusion criteria. By doing so, patients who were discharged from an ED with antibiotics for an unrelated indication were excluded. Second, data collection was limited to information available in the EHS due to the retrospective study design. For these reasons, adverse side effects were not collected as an outcome. Third, patients included in the study had low rates of pyuria. Another limitation is the different periods of the preimplementation and postimplementation groups because patient volume and staffing in the ED can be affected by different periods of the year. Additionally, patients were only called in the postimplementation group to clarify whether they had symptoms if it was unclear based on review of the EHS, which has the potential to introduce recall bias. Patients were not called in the preimplementation group, so determining presence of urinary symptoms was based on review of the EHS alone. Lastly, education on the new initiative was only provided to the ED providers and follow-up team. Due to the availability of culture data in the EHS, patients were able to see their urine-culture results and reach out to their primary care provider or urgent care providers for a prescription.

Although we were able to successfully utilize follow-up registered nurses as steward extenders to reduce the number of antibiotics prescriptions for ASB after discharge from the ED, there are several considerations for further improvement to reduce antibiotic prescribing for ASB. Urine analysis reflex criteria should be re-evaluated because the absence of pyuria has a negative predictive value of nearly 90%.^17^ In our current study, many patients did not qualify for treatment due to their white blood cell count. Optimizing the reflex criteria could improve ASB treatment rates across the hospital system. In addition to finding ways to decreasing urine culture ordering. Another future step of the initiative includes extending education on appropriate treatment of ASB to primary care providers.

In conclusion, implementation of an ASB assessment protocol for patients discharged from the ED significantly reduced the number of antibiotic prescriptions for ASB on follow-up calls and total antibiotic duration of therapy without an increase in 30-day admissions, 30-day ED visits, or 30-day UTI-related encounters. These findings demonstrate a process to utilize ED follow-up nurses as steward extenders to reduce overtreatment of ASB in a community health setting where resources are limited.
