# Implementation of an asymptomatic bacteriuria assessment protocol for patients discharged from the emergency department – CORRIGENDUM

**DOI:** 10.1017/ash.2024.65

**Published:** 2024-04-18

**Authors:** Margaret R. Hitchins, Jeannette L. Bouchard, Christopher W. Ingram, Alison I. Orvin

The sentence in the abstract results “There were significantly fewer antibiotic prescriptions for ASB in the postimplementation group (50% vs 87%; P < .0001).” should read “There were significantly fewer antibiotic prescriptions for ASB in the postimplementation group (50% vs 8%; P < .0001).” as stated in the body of the article.

The original article has been updated.
